# Effects of mental health stigma on loneliness, social isolation, and relationships in young people with depression symptoms

**DOI:** 10.1186/s12888-023-04991-7

**Published:** 2023-07-21

**Authors:** Katie Prizeman, Netta Weinstein, Ciara McCabe

**Affiliations:** University of Reading, Reading, England

**Keywords:** Depression, Mental health stigma, Loneliness, Social isolation, Subjective experiences, Young people, Qualitative research

## Abstract

**Background:**

Major depressive disorder (MDD) is the most prevalent affective disorder and the leading cause of illness and disability among young people worldwide. Besides being more susceptible to the onset of depression, young people have a higher risk of loneliness, and their personal and social development is impacted by social relationships during this time. It is thought that mental health stigma can undermine both help-seeking and longer-term outcomes for disorders like depression in young people. However, how stigma (i.e., related to depression) might affect young people’s feelings of loneliness, social isolation, and relationships is unclear. Using qualitative research methods, this study aimed to explore the subjective experiences of public and internalized stigma and its effects on loneliness, social isolation, and relationship quality in young people with depression symptoms.

**Methods:**

We carried out in-depth, semi-structured interviews with *N* = 22 young people aged 17–25 (M_age_ = 22 years) who reported high symptoms of depression (Mood and Feelings Questionnaire (MFQ) score > 27) (i.e., community sample, *N* = 9) or had been previously diagnosed with depression by a medical professional (i.e., clinical sample, *N* = 13). Data were analysed using thematic analysis. We explored the subjective effects of depression stigma on loneliness, social isolation, and relationships.

**Results:**

Participants described both public stigma (i.e., initiated by others) and internalized stigma (i.e., self-imposed) as disrupting social relationships and eliciting loneliness, isolation, and depressive symptomology. Four main themes about young people's subjective experiences of stigma were identified: 1) *Others’ Misunderstanding of Mental Health Disorders and the Impact Misunderstanding has on Relationships*; 2) *Effects of Stigma on the Self and Wellbeing*; 3) *Stigma Fosters Secrecy Versus Disclosure*; and 4) *Stigma Increases Loneliness Driven by Avoidance of Social Contexts*.

**Conclusions:**

Young people's accounts revealed a wide range of consequences beyond their depression diagnosis. Participants often felt discriminated against, misunderstood, and judged by others as a result of public stigma; they discussed internalizing these attitudes. They suggested that a lack of understanding from others, for example from their partners, family, and peers, and unreliable and/or absent support systems resulted in increased feelings of loneliness and social isolation and reduced the quality and quantity of relationship formation, social bonds, and interactions. Stigma also reduced their self-esteem and confidence, which in turn fostered secrecy and a reluctance to disclose their depression. Despite depression's stigma, most participants reported having long-term goals and aspirations to reconnect with others. These goals stood in contrast to feeling hopeless and unmotivated during periods of depression. Overall, we reveal how stigma can impact feelings of loneliness, social isolation, and relationships among young people with depression, which could lead to targeted interventions to lessen the impact of stigma in this population.

## Background

Major depressive disorder (MDD) is the most prevalent affective disorder and the leading cause of illness and disability among young people worldwide [18, 81, 71, 92, 95]. Young people aged 17 to 18 had a lifetime prevalence of 15.4% [71], comparable to that of young adults and older people, who showed a median 12-month prevalence estimate of approximately between 4 and 7.5% [4]. Besides being considered a major disease burden [100], MDD is also linked to long-term vulnerability, which may negatively affect young people's development [36, 101]. Young people who have experienced at least one depressive episode are more likely to experience long-term adverse health, economic, and social impacts [17], increased hospitalizations, suicide rates [48], and high rates of recurrence during adulthood [33, 35, 39]. There are direct links between youth depression and subsequent mental health difficulties [68], but it is also clear that the social context matters: the extent to which young people with MDD are supported by others influences the impact of the diagnosis on their mental health and wellbeing over time [56, 65]. It is imperative to understand why this might be and how we can encourage young people to seek help and find support.

Stigma may undermine mental health and wellbeing over time through its detrimental effects on social relationships; we posit that this is a key concern for understanding stigma in depressed youths. In addition to being more susceptible to depression, young people also have a higher risk of loneliness [1]. Loneliness develops when there is a perceived imbalance between desired and actual social connections [66], which impacts young people’s quality of life, illness prognosis, and willingness to seek care [86]. Whereas solitude suggests a desire to be alone and is fundamentally neutral, both loneliness and social isolation for extended periods of time are distinctly negative experiences [30]. Notably, individuals in solitude do not inevitably experience loneliness [70], and lonely young people do not always spend less time with other people compared to less lonely people [14].

Previous quantitative work suggests links between loneliness, social isolation, and depression in the younger population [41, 70]. However, there is a little qualitative knowledge of how these influence one another [1] and whether or not stigma experiences play a role in various aspects of relationships, including loneliness, social isolation, and withdrawal [37, 59, 96].

Stigma about mental illness is a broad concept comprised of negative stereotypes, prejudices (endorsement of stereotypes and emotional reactions), and discriminatory behaviours against people with mental health problems [22, 85]. It can be understood when a person has lost status in society because of their mental health [26, 42, 51]. Stigma involves feelings of humiliation—that one is at fault, degraded, or the object of displeasure [3, 54, 106]—and leads to feeling devalued, rejected, and stressed [31, 77].

When discussing stigma associated with mental health disorders, it is important to distinguish between two major forms of stigma: 1) public stigma and 2) internalized stigma [8]. Public stigma is when large groups of people in Western societies agree with negative stereotypes and act against or devalue people from certain social groups or classes, like those with mental health disorders [20]. Internalized stigma is when people have negative thoughts, beliefs, and stereotypes about their own conditions [21, 24]. It is by becoming preoccupied with society’s prejudices and/or hostility that mentally ill individuals internalize these objectionable stereotypes [11].

Youth is a time for exploring oneself in relation to society and social contexts, as well as a key period for individuals to form strong relationships [27, 49]. In the case of mental health stigma, young people with mental health difficulties may struggle to incorporate these experiences into their identity or to share these aspects of their identity with others for fear of rejection or hostility [12, 72]. Previous research has shown that young people are subject to stigmatizing contexts and experiences that may be unique to their particular developmental challenges [74]. For example, the transitional period from childhood to adulthood is commonly known to be a sensitive period in which lived experiences can have profound long-term effects on future health and development [52, 91].

Young people with depression must deal with those symptoms, but they are also challenged by associated stigma and possibly linked feelings of loneliness and isolation [23, 88]. Stigma has been identified as a factor in the negative outcomes and wellbeing of young people with MDD [24, 74]. It is possible that the reduced quality and quantity of relationships and social interactions, or lack thereof (i.e., social withdrawal), that are apparent in depression [65] could in part be due to stigma rather than the depression itself [12, 72]. In particular, studies find that public and internalized stigma can negatively impact young people's development of a sense of self (i.e., self-identity, self-esteem, and self-worth), social relationships, and withdrawal [53, 93]. Research into mental health stigma has largely focused on investigating this concept in adults [74], much less is known about young people with depressive symptoms and the stigma they experience [50, 67, 94]. More research is needed to better understand public and internalized stigma in these populations [32, 38]. In particular, how stigma influences the loneliness and social relationships of youth depression is important to understand, as young people are at a developmental stage where social connections are key [81, 84].

Research on how mental health stigma is experienced by young people with depression is limited [74], as is research on its impact on loneliness, social isolation, and relationships [69]. Taken together, conceptual frameworks are needed that describe how young people experience depression stigma and its impact on loneliness, social isolation, and relationships, given that these social experiences and emotions are key factors that influence young people's development into adulthood and that could worsen mental health outcomes [19, 81].

In this study, we explore the subjective experiences of mental health stigma and its influence on feelings of loneliness, social isolation, and relationship experiences. To address research gaps, this study focused on two main research questions: 1) What are young people's subjective experiences of mental health stigma? and 2) How does stigma influence feelings of loneliness, social isolation, and relationship experiences in young people with depression symptoms? The age range of 17–25 years was selected to better understand this key early age in adult development that reflects the transition from adolescence to adulthood [61, 87]. This age range focuses entirely on the younger population, also referred to as "young people".

This study's major aims are to establish the existing literature among young people in order to better understand their subjective stigma experiences as well as explore any effects stigma may have on experiences of loneliness, social isolation, and relationship experiences. To provide for flexibility and give young people a voice, semi-structured interviews with a number of open-ended questions were used. These questions allowed participants to direct a discussion based on personal experiences of stigma. Due to its more open-ended character, this interview technique adds more detail and richness, enabling us as researchers to identify patterns while still allowing comparisons between participants [58]. The primary objective of our approach is to provide young people's subjective experiences to inform the development of stigma research and awareness in order to prevent or minimize adverse stigmatizing experiences in young people with depression. Understanding young people's experiences with stigma and how it affects feelings of loneliness, social isolation, and relationships may help develop more effective targeted interventions that reduce stigmatizing attitudes and beliefs about depression.

## Methods

### Participants and recruitment

Participants' demographics and clinical characteristics are presented in Table 1.

**Table 1:** Participant demographics and clinical characteristics

**Participant**	**Age**^**a**^	**Gender**	**Ethnicity**	**Country**	**Education level**	**MFQ scores (/66)**^**b**^	**Sub-sample**
P01 P02 P03 P04 P05 P06 P07 P08 P09 P10 P11 P12 P13 P14 P15 P16 P17 P18 P19 P20 P21 P22	21 21 24 25 17 22 20 25 22 25 25 25 25 21 25 21 22 21 19 22 21 17	Male Female Female Male Male Female Other Female Female Female Female Female Male Female Female Male Male Male Male Female Male Female	Asian White White Black/African American White White Mixed/Multiple Ethnic groups Asian White White White Black/African American Black/African American Asian White Black/African American Black/African American Asian Asian Black/African American White Black/African American	United Kingdom United Kingdom South African United Kingdom United Kingdom United Kingdom United Kingdom China United States of America United States of America South Africa South Africa Wales Philippines South Africa South Africa South Africa India South Africa South Africa United Kingdom United Kingdom	University University University University Sixth form University University University High school University University University University University University University University University University University University Sixth form	50 35 13 38 28 56 27 18 47 58 14 63 34 62 44 27 46 39 64 27 47 18	Clinical Community Clinical Clinical Community Clinical Community Community Clinical Clinical Clinical Clinical Community Clinical Clinical Community Clinical Community Clinical Community Clinical Community

*MFQ* Mood and Feelings Questionnaire (higher scores indicate more depression). All participants completed the long version of the MFQ

^a^Age at interview

^b^MFQ score at screening or diagnosis

Participant recruitment from the community involved emails to listservs, and the study strictly followed the standards of voluntary and informed consent and data protection for each participant who voluntarily fulfilled the inclusion criteria. Participants included 22 young people between the ages of 17 and 25 (*M* = 22 years) who had high symptoms of depression or had been currently diagnosed by a medical professional. Fifteen of the 22 participants took part in the interview. There were no other inclusion or exclusion criteria. Depression symptoms were assessed through a pre-screen using the Mood and Feelings Questionnaire (MFQ) [25] with a benchmark score of > 27, administered by the primary researcher (KP) via an online survey prior to individuals taking part in the study. Of the sample, *N* = 13 had a current diagnosis of depression by a medical professional (Table 1).

## Data collection

### Mood and Feelings Questionnaire (MFQ)

The MFQ is a 33-item scale that measures depressive symptoms in children and young adults. It has good psychometric properties [29, 83]. Participants' responses indicate how they have been feeling or acting in the past two weeks [25]. A cut-off score of 27 and above has been identified as the difference between clinical and non-clinical levels of depressive symptoms [105]. Each item is rated on a three-point Likert scale from 0 (*not true*) to 2 (*true*). This questionnaire is widely used to score depression in young people, with higher scores suggesting more depression symptomatology [105].

### Semi-structured interviews

Using their clinical and research expertise in the fields of mental health stigma, MDD, loneliness, social isolation, and relationship experiences, the authors created a semi-structured interview schedule to guide discussions (Table 2). Participants completed demographic questions regarding age, gender, education, geographical location, and ethnicity prior to semi-structured interviews. Semi-structured interviews (*N* = 22) were scheduled and conducted online (using the Microsoft Teams platform) with participants by KP between June 2022 and August 2022 (post-COVID-19 lockdown). Interviews were conducted until data saturation was reached, meaning that no new information was observed and collected [46]. Interviews were audio-recorded, transcribed verbatim, checked for accuracy, and subjected to thematic coding by KP. Each interview lasted between approximately 25 and 60 min and was conducted in English.

**Table 2:** Qualitative interview guide

**Focus Area**	**Sample Questions and Probes from Interview Protocol**
**Demographic Questions:**	Age: Country: Education level (i.e., School/University): Ethnicity: Gender (Male/Female/Prefer not to say/Other):
**Broad/General Mental Health Stigma Experiences:**	1. Have you ever experienced stigma – unfairness, bias, or discrimination because of your mental health? Can you describe how you first became aware of being stigmatized and what the experience was like for you? 2. How do you feel you’ve been affected by public-, or internalized stigma with regards to your mental health? Please explain how these two types of stigmas have affected you, for example, have they influenced your thoughts, feelings, or relationships with others. *If mentioning public but not elaborating*, could you tell me more about how public stigma has affected you? *If mentioning internalized but not elaborating*, could you tell me more about how your internalized stigma has affected you? 3. Who do you talk to about your mental health? Please explain your answer How do these people react to your mental health condition? How does this make you feel? How does the experience of stigma impact your relationships with your family? How about with your friendships? 4. Who do you not want to talk to about your mental health? Why? How does it feel to hide your mental health (i.e., add stigma/psychological pain)? 5. Have you ever talked about your mental health online? How has being online changed your experience of mental health stigma?
**Specific Event Recalled:**	6. Can you tell me about a specific time you felt stigmatized? What was it like for you? Was it public or internalized stigma? 7. Thinking back, how does the experience you have had influence the way that *you* socialize (i.e., spend time with others or spend time alone)? How has this experience changed the way you feel around people with a mental health condition? How has this experience changed the way you feel around people without a mental condition? How does this specific experience influence your time alone?
**Recovery Questions:**	8. Thinking about stigma is there anyone (i.e., friend, clinician, guardian), or anything (i.e., self-help book, impactful mental health experience, mental health website or forum) that has helped you cope with your mental health? Are there ever times you consider not doing these because of the opinions of other people? Have other people protected you from feeling mental health stigma? If so, can you give an example? How has this influenced the way you think and feel? 9. In your opinion, what do you think should or could be done to prevent both public and internalized stigma, if anything? 10. In your experiences, what advice can you give others also living with a mental health condition? How do you think it is best to deal with stigma? Or how would you help others experiencing mental health stigma? 11. Is there anything else you would like to share?

While some of the semi-structured interview questions mentioned direct concepts such as stigma, loneliness, and social isolation, other questions were more open-ended, allowing participants to describe these organically. Interview questions were piloted with young people aged 17–25 and revised as necessary. The topic guide explored the following: 1) broad/general mental health stigma experiences; 2) specific events recalled; and 3) recovery questions. Recovery questions were used to give participants an opportunity to talk about any other opinions or experiences they would like to share. See Table 2 for a summary of the qualitative interview guide.

The topic guide was used flexibly through a semi-structured interview and comprised interview questions relating to subjective experiences of mental health stigma, followed by prompts to gather richer data about each individual’s particular experience. The first author (KP) conducted all the interviews online. They took place online using the Microsoft Teams platform, in a quiet room with only the researcher and participant present. Interviews were audio recorded and lasted an average of 30 min (range 20–50 min). Participants were placed into a draw to receive a £40 gift voucher for their participation. Interviews were conducted until saturation was reached, such that the data collection process no longer offered any new or relevant insights [46]. Interviews were transcribed verbatim by KP. Field notes were made after the interview and used to aid analysis.

## Data analysis

Thematic analysis (TA) was employed to identify and analyse patterns of meaning in the dataset. This technique is ideal for investigating how a group conceptualizes a particular phenomenon [47]. TA is not tied to a particular ontological or epistemological position,therefore, in this study, the researchers adopted a post-positivist critical realist stance [44]. This position assumes that reality is observable and quantifiable while recognizing that participants are unaware of all the factors that influence their experience [47]. Furthermore, to align this work with emerging literature on stigma experienced by people with mental health problems, we chose a pragmatist approach to materials and an abductive process of data analysis (i.e., inference to the best explanation allows inferring, such as "a" as an explanation of "b") [57]. Primarily, we used an inductive, “bottom-up” approach [78]. Without first attempting to fit the data into pre-existing coding schemes, the data were examined. That being said, we did not dismiss possible themes that did not fit current literature, nor did we identify themes unless they were evident in the data.

The researchers considered their own sources of bias and prior assumptions, including knowledge of depression and mental health stigma (KP), when conducting research into young people’s mental health (KP, CM, NW). The data were analysed using constant comparative techniques based on Braun and Clarke's six-stage TA method [10]. In stage 1), the first author (KP) familiarized themselves with the data by conducting and transcribing interviews and then rereading the transcripts. In stage 2), KP conducted line-by-line coding. The process of coding was inductive and iterative, with constant comparisons between and within transcripts. Initially, all data were coded for both explicit and implicit meanings. The labelling of codes focused on capturing the subjective experiences of mental health stigma as well as its implications for feelings of loneliness, social isolation, and further wellbeing concerns [55, 97]. In stage 3), codes were combined into potential themes that reflected the data's major characteristics and patterns. In stages 4) and 5), themes were evaluated by examining all codes and themes in aggregate [10, 45]. Tentative themes were reviewed by the research team (KP, CM, NW) [79, 102]. During these coding meetings, alternative interpretations of patterns in the data were considered and discussed until a consensus was reached. In the final stage 6), themes were finalized, and quotations exemplifying each theme were identified.

## Results

See Table 3 for a summary of the themes discussed below. Similar viewpoints were shared by those with and without a formal depression diagnosis.

**Table 3:** Table of themes and sub-themes

**Themes**	**Sub-themes**
1. Others’ Misunderstanding of Mental Health Disorders and the Impact Misunderstanding has on Relationships	a. Hallmark/Typical Symptoms Associated with Depression b. Understanding Acts as A Support System
2. Effects of Stigma on the Self and Wellbeing	a. Diagnosis and Self-Identity b. Getting Out of the "Depressed Rut"—Self-reliance, Self-awareness, and Dispositional Optimism
3. Stigma Fosters Secrecy Versus Disclosure	a. Suffering in Silence – The Choice of Secrecy b. Seeking Disclosure for Freedom
4. Stigma Increases Loneliness Driven by Avoidance of Social Contexts	a. Choosing to Spent Time Alone b. Seeking Alone Time for Purpose c. The Desire to Socialize

### Overview of themes

Young people’s subjective experiences were captured in four main themes: 1) *Others’ Misunderstanding of Mental Health Disorders and the Impact Misunderstanding has on Relationships*; 2) *Effects of Stigma on the Self and Wellbeing*; 3) *Stigma Fosters Secrecy Versus Disclosure*; and 4) *Stigma Increases Loneliness Driven by Avoidance of Social Contexts* (Table 3). Each theme highlighted a unique aspect of stigmatizing experiences; however, there were areas of conceptual overlap between themes. All major themes and sub-themes were expressed by both those with and without a depression diagnosis. Participants also expressed that internalized stigma was an antecedent to bigger problems, such as a lack of honesty and disclosure in relationships, poorly formed relationships, difficulties trusting and communicating, a negative self-identity, and low self-esteem. We explore these below: All themes were further defined by sub-themes that depicted the nature of conversations (Table 3).

## Theme one: Others’ misunderstanding of mental health disorders and the impact misunderstanding has on relationships

During interviews, participants talked about how most of the people they had a relationship with did not understand their mental health conditions or what it meant to have a mental disorder. They said this affected their relationships and social interactions.

### Sub-theme: Hallmark/typical symptoms associated with depression

*“...when people experience depression, they kind of have very hallmark typical symptoms that people associated with it.”* (P11, 25, Female)

Participants said that most people have a set idea of what depression is and what the common symptoms and stereotypes are. One of these ideas is that others think that depressed people are sad and suicidal. Participants also talked about how others see, talk about, and try to discredit MDD (for example, by claiming that MDD is not a real medical condition or is "nonsense"). Participants expressed that these stereotypes made them feel judged, undervalued, and misunderstood by others.

Participants felt that others did not understand what it meant to be diagnosed with depression or the signs of MDD. Participants expressed negative emotions such as shame and guilt as a result of others’ lack of understanding, and they reported that such misunderstanding negatively impacted the way they saw themselves (i.e., they experienced lowered self-confidence, self-esteem, and self-worth, as well as insecurities such as feelings of abnormality and weirdness). Participants knew that others cared for them but lacked education about, e.g., depression. Still, the majority of participants would choose not to invest in relationships with those who misunderstood them, as they felt that it would do more harm than good.

*“So, like, I have this one friend. He doesn't have any mental health issues, but like when I talk to him, he, like, just doesn't quite say the right thing. But it's like, I know that that's just because he doesn't understand. Not because he doesn´t care.”* (P02, 21, Female)

*“...I've made that conscious choice to spend time with people who do care and at least try to understand.”* (P02, 21, Female)

Participants reported that they were sometimes unable or found it difficult to make friends or communicate with others due to thinking or feeling that they were being judged by those around them. This made participants feel that others viewed them as crazy and abnormal, views they also came to believe (i.e., internalized). Once alone with their thoughts, some recognized that these thoughts and feelings may be a protection mechanism as a way to guard oneself from judgment, stigmatizing experiences, and stereotypical attitudes and beliefs. By avoiding getting close to others or forming relationships, participants were unable to experience victimization and also avoided the topic of mental health coming up in discussion. Some participants chose to continue isolating because it felt less painful than worrying about what others thought.

*“And then, as you get to know each other more, you realize that it’s more and more important to talk about the way you feel. When that happens, your relationship inevitably changes. That’s quite hard…”* (P21, 21, Male)

### Sub-theme: Understanding acts as a support system

Some chose to talk about their mental health diagnosis with others with whom they felt comfortable and who had had similar experiences with depression. They did this to fight the silencing effects of stigma and to paint a more accurate picture of what it means to be diagnosed with depression. However, some felt that their self-disclosure furthered their own guilt and others’ blame for their mental illness, which in turn led to increased internalized stigma, secrecy, and self-identity problems, including low self-esteem. Such conversations created a further feeling of being stigmatized.

*“...it is definitely still challenging talking to people about things like self-harm and what that means to people, I think is quite misunderstood. And with stigma, you just sort of... There’s a lot of stigmas there, really. And yeah, I think that’s definitely affected me as well.”* (P06, 22, Female)

Participants also described having a positive social experience when they shared aspects of MDD. For instance, participants describe the positive influence sharing their experiences has if it is with others with similar experiences of MDD. They describe the positive effects of people who show care and support for individuals with mental illnesses and who have a better understanding of mental disorders. Participants expressed the positive influence that understanding, support, and care had on relationship bonds and their associated social interactions, as well as how they positively impacted their self-worth and wellbeing.

*“Yeah, kind of because I feel like most of my friends have similar issues. So, I feel generally more understood. When I’m at home, I kind of just want to go be alone because I feel like people just don´t understand as much.”* (P02, 21, Female)

*“So, my mother, she takes it very personally because her mother was also diagnosed with depression and she committed suicide* [sic]. *So, my mother, it like scared her the first time I told her that I was diagnosed, but she was still very supporting… And my sister, she struggles with generalized anxiety disorder, so I think, she and I, we um, we support each other with medications. Um, like she is starting with new antidepressants now. So, we just we support each other. We like talk to each other, like, kind of experiencing kind of experiencing kind of the same thing in a way.”* (P03, 24, Female)

## Theme two: Effects of stigma on the self and wellbeing

Participants expressed that having a formal diagnosis made it harder for them to shake the feeling that they were not good enough or useful. Participants felt this unintentionally changed how they saw the “self” (i.e., lowered self-confidence, self-esteem, and self-worth), as well as the way they responded to being in social contexts. The majority of those interviewed stated that their own stigmatizing experiences started out in public and gradually became internalized over time.

*“I tend to actually believe it when it’s stuff like that. It's more when they say things like, ´That's stupid for thinking that sort of thing.´ I think it´s supposed to be reassuring, like ´Oh, you don´t need to worry,´ but it´s kind of the way they say it, ´Ah, why you´re being so stupid for thinking that sort of thing?´ So yeah… stuff like that.”*(P02, 21, Female)

### Sub-theme: Diagnosis and self-identity

Participants expressed experiencing a decrease in self-esteem and self-worth after being diagnosed with a mental illness. Many participants said that their first diagnosis was a turning point in how their identity changed. Participants' self-destructive views and thoughts of the "self" began with feelings of weakness and insignificance, increased guilt, and a lack of empathy and kindness.

*“I think the fact that before I was diagnosed, I also thought it was just a bunch of nonsense. I think in the beginning I was quite mad, um, I was dealing with it for such a long time, and I was also just thinking that this is nonsense. You know, when I was diagnosed, I was like, ´Okay, I’m one of those people now.´”* (P03, 24, Female)

*“It made me feel like I am the problem. I’ve always thought like I´m a problem and having someone else confirm it just made me feel worse because I try so hard, but at other times really, I struggle.”* (P17, 22, Male)

A person's motivation, mental health, and overall quality of life can all be hurt by low self-esteem [5, 34]. These key elements were expressed as negatively influencing one’s self-worth, self-esteem, and wellbeing and were recurring themes in participant interviews. Participants experienced decreased self-confidence, feelings of insecurity, a lack of identity, and feelings of not belonging.

*“It certainly affects your self-esteem… there’s definitely something like that constant, almost like rumination of like, ´Are they judging me? Am I behaving normally? Like, are they gonna judge me if I cancel this because I am not feeling up to it?´...that kind of stuff.”* (P11, 25, Female)

Only a small number of individuals took something positive out of receiving a diagnosis, such as unexpected insights and an appreciation of how essential one's mental health is to one's identity. Feelings of relief and recognition were also mentioned after receiving a diagnosis.

*“Yeah, I think for myself, I felt that I wasn't as strong as I thought I was. Because you know, I had like this sense of you know, I have to be strong to deal with things like my life is really not that bad. And then when I was diagnosed, I was like, I felt relieved and significant in a way. Like I´m not crazy for feeling that way.*” (P09, 22, Female)

*“No, um, because when I was diagnosed, initially, five years ago, um, I think I did at that point because it was just after I was diagnosed that I decided to study psychology. And I think before that, I was also under this impression of, ´Yeah it´s all just in your head, you should be strong´, and um, that kind of stuff. I think the internalized stigma I probably experienced at the beginning, like very beginning, but it was just a few months after I was diagnosed, that I started to realize that, ´Okay, this is real, this isn't just me being I don't know a little baby or whatever. ´ Actually having a diagnosis has taught me a lot. That´s something I wouldn´t have expected and yeah it had made me who I am essentially.”* (P10, 25, Female)

### Sub-theme: Getting out of the "depressed rut"—self-reliance, self-awareness, and dispositional optimism

Many participants credited their mental wellbeing to inborn or learned independence or confidence. Others mentioned gaining these attributes in early adulthood (for example, through life experiences). Participants described these attributes as helpful in that they were better able to ignore stigmatizing attitudes and beliefs around their mental health diagnoses. These participants had lower internalized stigma because they rejected social messages; they attributed this capacity to higher wellbeing.

Participants stressed the importance of maintaining a positive attitude in order to avoid becoming permanently depressed. Most participants expressed that having a set routine is helpful in gaining a sense of contentment, while being self-aware of the pre-stages of a “rut” can aid in an optimistic attitude. Some participants were able to implement a mindset of looking for happier life experiences despite their mental health conditions.

*“For me the first step is, the easier you can cast it out of your mind, as a first step, the happier you will be already… Once you have that solidified, I think it is much easier to then think you know, ´There’s no reason for me to care what they think of me. There’s no reason for me to let that take up so much space in my mind. ´ And as a consequence of that, chances are you won’t be stigmatized as much because people will see that you're in more control.”* (P21, 21, Male)

## Theme three: Stigma fosters secrecy versus disclosure

Disclosure of mental illness was commonly identified as being based on the trust, understanding, support, and care given by the person they are sharing their feelings with. Some participants who were not always able to hide their conditions due to physical signs and symptoms (i.e., incisions and scars) found the idea of secrecy and/or lying difficult. Still, many who took part said that they do not talk about their mental conditions. Participants stated that keeping mental illnesses a “secret” was done to avoid potential frustration and disappointment from others’ lack of understanding and support. Participants suggested that talking about their mental illnesses would lead to more adverse outcomes (i.e., further misunderstanding and stigmatization), which would hurt rather than help their overall wellbeing.

*“...sometimes I feel like I'm being a bit dishonest and like if I'm hiding something and it makes me feel bad if ‘m not being honest. But other times, it just makes me feel frustrated because I feel like I can’t tell them.”* (P02, 21, Female)

*“Generally, I will be open with people even if I don’t particularly want them to know because a lot of the time, I feel like I can’t hide it. I have like, visible scars on my arms. So, I feel like it’s really hard for people not to know most of the time, and so I sort of feel like, yeah, just be as open as I can.”* (P03, 24, Female)

*“By not talking about it [I feel protected] ...It means that most times, I can pretend that I don’t have a problem and can´t disappoint people counting on me…”* (P15, 25, Female)

### Sub-theme: Suffering in silence – the choice of secrecy

Participants described not wanting to share mental health details as a way to avoid feelings of weakness, incompetence, and insignificance. The majority of participants thought it would be ideal to suffer in silence rather than be a burden to others.

*“I really don’t want to talk about it... I just feel like it’s something like I’d rather not acknowledge that I have. So, I’ll tell people, ´Oh no, I’m just having a bad day.´ But there are times where I would like to talk to people but I just cry myself to sleep alone and then wake up pretend everything is okay. I really don’t like talking about it. I can´t say for sure why. But maybe it’s because I don’t want people to judge me, but I don’t like talking about it at all. Not with anyone.”* (P17, 22, Male)

Participants said that one way to avoid talking about mental health issues is to lie about them. This was also said: it is their first instinct to lie when trying to avoid the topic. Participants also talked about hiding medications or taking them in private. This was done so that no one else would ask questions that would lead to a conversation about mental health.

*“...Also, I had to hide my antidepressants that I got from the doctor this year while I was taking them, because I don’t take them all the time, just when I’ve been prescribed. So, I thought I had to hide them to avoid people asking what´s wrong or why am I taking medication or something.”* (P15, 25, Female)

### Sub-theme: Seeking disclosure for freedom

Disclosure was recognized as a helpful part of expressing one’s thoughts and feelings—to those with whom participants trust, feel better understood, and feel unjudged. Participants gave numerous accounts of how they told others about their mental health problems because they thought it would help build trust, strengthen relationships, and give them more freedom.

*“Um, it´s not as if you´re off-loading onto them, trying to give them the problem. You´re just being honest, which I guess is a good first step if you want to grow and be more mature for me anyway.”* (P01, 21, Male)

*“I feel like being open about mental health issues, um, just shows other people like, that’s okay... And they start to realize maybe there’s something I need to deal with... So, I think just talking about it and just breaking the stigma just helps people with, um, underlying disorders in general.”* (P03, 24, Female)

## Theme four: Stigma increases loneliness driven by avoidance of social contexts

Participants with depressive symptoms were asked to discuss how they manage social situations and the uncomfortable emotions that can arise when around other people. Many participants shared that in order to avoid these kinds of situations and, as a result, unpleasant emotions, they would prefer to be alone.

### Sub-theme: Choosing to spend time alone

Some participants expressed feeling grateful to have time to themselves and said that they would like to be around other people if they did not have to deal with discrimination. The majority of the participants said they were aware that when they were alone and did not have any outside interactions or distractions, they may be inclined to reflect on negative thoughts and feelings.

*“I prefer to spend time alone so that nobody will look down on me and nobody will stigmatize me. So, I prefer staying all alone…”* (P13, 25, Male)

### Sub-theme: Seeking alone time for purpose

Many participants identified time alone as a means to fulfill a number of purpose-oriented goals. This time alone allowed them to feel still and peaceful, revitalized and restored, and to reflect on their past week. As a result, they felt more confident in themselves. Further goals included doing simple activities they enjoyed (i.e., reading), making sense of things, or finding meaning in life.

*“Often, I think when I look back and how my week went and how I spend my evenings, I definitely could have planned in a couple of times where I could have tried to see more people. That wouldn´t have affected me negatively at all. If anything, I would have made me feel a bit better. Take what I feel I´ve learned and put it into practice. Otherwise, you're just stagnating, and you get caught in the same cycle. I think you can make other people just as much a part of your life just as much as you make yourself. Like it's my choice, making my life just about me.”* (P01, 21, Male)

*“So sometimes I prefer to spend time with like my close-knit circle. Um, but I'm also very comfortable being alone. But um like I normally use my alone time for quite a bit of reflection, and yeah, I find this really helps, like it's good for my mental health.”* (P07, 20, Other)

### Sub-theme: The desire to socialize

Being around other people did not relieve feelings of isolation and aloneness, but in addition, participants shared that alone time did not help improve their mental health. The majority of participants expressed that being alone was not always their desired choice but rather a consequence of being mentally drained in social situations. For example, constantly worrying about what other people think of you and how they will react to or evaluate specific things you say or do. As a result, stigma may be exacerbated or introduced.

*“It takes effort, I find, to talk to some people. And often it takes effort, mostly because of how critical I am of the way that I am talking. I am constantly thinking about what will be the best thing to say in this situation. What will they think of this if I say this, etc.? And that can be quite tiring, and I find that, especially with a lot of my friends who are quite introverted and have a social battery, you can see the exact moment when it runs out and they want to be alone, and um...You know what I mean? I mean, like… if I had the choice, it would be to be around my friends and to get out more. But yeah… So, it is hard to say that being on my own helps. It is just something that, at times, I need to. It´s more a consequence of being tired. And I think I am not going to be of any benefit to anyone if I talk to them in this state, so I am just going to relax and do my own thing. So that´s often the thought process behind being alone, I think.”* (P01, 21, Male)

*“So, I think that it definitely brings in that isolation factor of, like, you know, it's difficult to make friends because there's this constant voice in your head saying like, ´You're not worthy. People don't like you´, and like all of that. So then, you´re kind of, like, it's difficult to like make friends or make meaningful connections or like get out of your head enough to put yourself out there even though you really want to.”* (P14, 21, Female)

For some, socializing is related to the desire to show others a “better” version of themselves, while others say they use socializing as an agent of distraction or numbness.

*“Yeah, I think even if I’m talking with them happily and they think I am outgoing. But I think they don’t know me. Yeah, I’m still alone. Just, uh... It’s my mask or it’s my uh, uh, uh outside expression… Although like it might be false, at least I get to portray a better person...Specifically with depression, I’d say that you probably find life easier if you just try to fake it till you make it around other people. Whether that’s the right thing or not to do, you'll find life easier if you fake it till you make it.”.”* (P19, 19, Male)

*“I prefer to spend time with others because it’s sort of like a distraction and a numbing agent. I get to, like, I get to only portray the side of myself that makes me happy, I guess.”* (P19, 19, Male)

That said, many participants were aware of the fact that socializing would be beneficial for their mental health despite existing stigmas, even if they did not necessarily feel like it.

*“Uh, these experiences, uh, personally, makes me to really think this is much better to be alone rather than when we are with people even though this doesn´t benefit me. I, I want to be social. I want to be around my friends. Uh… but you know anything can still happen and you still get victimized or, because uh… you know when I am with people I forget about the depression. But I can’t do this if I get victimized you know… It´s hard.* (P04, 25, Male)

## Discussion

There is a growing interest in developing evidence-based strategies to fight the impacts of stigma among depressed youths [32]. This qualitative study explored the subjective experiences associated with public and internalized stigma among young people with depression symptoms. It builds on the existing literature focusing primarily on deficits in knowledge about stigma and barriers to mental health care [32, 38, 50] and addresses the need for research on young people's subjective experiences of stigma and its effects on a number of emotional (e.g., loneliness) and functional (e.g., isolation) dimensions of their social relationships.

Our study suggested that mental health stigma continues to be a significant problem experienced by the younger population; most participant responses referred to having experienced externalized public stigma across their lifetimes. They felt judged, misunderstood, and insignificant. The vast majority of those described had internalized these stigmatizing experiences. Notably, the majority of participants described this process as occurring over time, stating that their experiences with stigma began in public and gradually became internalized.

We identified four main themes that reflected patterns in participant responses to interview questions, which highlighted that stigma’s negative effects on young people’s experiences of depression occur for varying reasons. Summarized in the first theme (titled *Others’ Misunderstanding of Mental Health Disorders and the Impact Misunderstanding Has on Relationships*), the young people with depression with whom we talked often felt misunderstood (i.e., the lack of and/or lack of understanding of friends and family, the support system, the formation of bonds, and the strengthening or weakening of relationships) and stereotyped by others due to their MDD, resulting in fractured relationships. *Effects of Stigma on the Self and Wellbeing (i.e., in the context of development, self-identity, wellbeing, and personality)* reflected our observations that stigmatizing experiences harmed participants' self-identity, influencing their self-worth, self-esteem, and overall wellbeing in negative ways. *Stigma Fosters Secrecy Versus Disclosure (i.e., mindset, circumstances, and experiences)* recognized that participants discussed predictors that caused them to feel embarrassed about their depression, leading to views that secrecy is necessary and inhibiting disclosure. On the contrary, participants also identified reasons as to why some individuals disclose their mental illness, such as because it provides a way to find comfort and closure.

In our final theme, *Stigma Increases Loneliness Driven by Avoidance of Social Context Bonds (i.e., loneliness, social isolation, and social circles)*, we identified that stigma related to MDD is an isolating experience that affects communicating with others, socializing, the formation of bonds, and increases the desire to be alone. Most participants had negative stigma experiences, which in turn led them to choose to spend time alone even when alone time was not a valued activity. Being alone was a tool for managing stereotypical attitudes and beliefs and avoiding further stigmatizing experiences within the social context. That said, many actively anticipated, planned for, or sought periods of alone time as a way to find balance with their noisy, outer worlds.

Our findings that *Others’ Misunderstanding of Mental Health Disorders and the Impact Misunderstanding Has on Relationships* are aligned with previous studies with young people [9, 16, 103]. They highlight the relevance of stigma for driving feelings of being misunderstood and judged and build on a body of work that has largely focused on lack of parental warmth and support and childhood maltreatment as early predictors of MDD (i.e., parent–child relationships, lower positivity, negative emotionality, and disconstraint) [80, 89]. Research has consistently shown that relationships between people and their effects on internal experiences shape development in complex ways and are likely to interact with one another to affect the onset of depression [80, 90].

Research findings have shown how crucial it is to make social support networks safer (e.g., by focusing on improving mental health understanding in relationships) in order to reduce the risk of depression and potentially treat it during the developmental phase, when depressive symptoms usually start to appear and peak [16, 43]. Interventions that emphasize supportive and understanding parent and peer relationships reduce depression symptoms [40]. In our sample, participants reported that their negative emotions (i.e., feelings of shame and guilt) deterred them from investing in relationships and taking part in social interactions where misunderstanding and stereotypes attached to depression are consequential factors. Such social interactions were seen to reflect a lack of understanding and interplayed with internal factors (i.e., effects on self-esteem, self-confidence, and insecurities) in determining whether young people with depression chose to invest time in these relationships.

Further reflecting on our theme, *Effects of Stigma on the Self and Wellbeing*, it is worth noting that stigma had an important influence on self-esteem and self-worth in our sample, an observation that echoes studies in young people [7, 73, 76, 82, 99]. Past research has also found links between experiences of mental health stigma and reduced wellbeing [28]. Our study showed that for many participants, having a formal diagnosis unintentionally changed the way one saw oneself (i.e., lowered self-confidence, self-esteem, and self-worth, as well as increased insecurities and a lack of identity). As a result of these self-processes, young people with depression were more likely to avoid social situations. They increased social withdrawal as a result of adverse self-views (i.e., poorer wellbeing).

It is worth noting that despite these costs, a few participants benefited from having a formal diagnosis, such as gaining unexpected insights and understanding the importance of one's mental health in accepting oneself (bringing about feelings of relief and recognition). This finding is consistent with past research, which has shown the importance of self in young people with depression [63].

Our findings that stigma fosters secrecy versus disclosure are in line with previous studies, which have found people keep their depression a secret because they feel ashamed. Furthermore, people with depression may be reluctant to acknowledge their illness due to the guilt and stigma associated with it [13, 104]. Past studies have identified that secrecy can be a way to protect against stigma despite its adverse long-term consequences, such as loneliness and social isolation [75]. In line with previous research, our study showed that for many participants, talking about depression led to more adverse outcomes (i.e., added stigma, stereotypical attitudes and beliefs, and increased judgement), thus limiting their desire to disclose depression [15].

In addition, our findings that stigma increases loneliness driven by avoidance of social contexts have been found in both adults and youths [6, 60, 62]. Our participants described why they chose to spend time alone. Specifically, they highlighted isolation as a way to avoid stigma and discrimination. Alongside this avoidance goal, time alone held some benefits for young people. Many participants expressed having this time to fulfill purpose-oriented goals as well as for reflection, revitalization, restoration, and pleasureful activities, such as reading. Results further indicated participants' desires to socialize, even though this did not alleviate feelings of loneliness. This was often used as a way to show others a better version of themselves and is in line with past studies [2, 98].

Given the importance that participants placed on feeling misunderstood or judged, it is important for practitioners who seek to help young people with depression to demonstrate understanding of mental health and stigma, as well as show empathy and care for depressed individuals' feelings and experiences. Doing so may have lasting positive impacts on wellbeing [16]. Through its findings, the current study extends the literature in several ways. While many studies have focused on stigma, our study explores young people's subjective experiences, an area in which research on the younger population is limited. Findings extend understanding of stigma effects, identifying their implications for feelings of loneliness, social isolation, and disrupted relationship experiences. Few studies, if any, have looked at all these ideas simultaneously in this population with an emphasis on depression.

## Limitations and future directions

The qualitative study provided rich data and gave voice to young people with a formal diagnosis of depression and those who reported high symptoms of depression. However, we only recruited one in-patient participant, and therefore, it is possible that the more severe cases of MDD were not captured in this study. Furthermore, it was difficult to tell which aspects of the participants’ experiences were due to stigma itself and which were due to the nature of MDD impact on wellbeing. Future qualitative and quantitative research could examine depressive symptoms and stigma side-by-side to identify their unique and dependent effects on social and emotional wellbeing.

Likewise, while there was some diversity among participants in terms of socio-economic status and gender, the study would have benefited from greater diversity in terms of geography and culture, as this will be important as we continue to seek an understanding of mental health stigma. Mental health is understood and received differently in various countries, and as a result, stigma may have diverse consequences across cultures [64]. That said, this study was not designed or equipped to disentangle culture from individual participant experiences, but we sought diverse views that reflected participants’ unique experiences within their different cultures. Future quantitative research can rigorously examine the extent to which the subjective experiences described by the participants in this study are representative of those of the larger population of young people exhibiting depressive symptoms within their cultural context, but future qualitative research can also explore with participants how they see their particular sociocultural conditions affecting stigma and its outcomes.

## Conclusion

Young people's accounts revealed a wide range of consequences beyond their depression diagnosis. Participants described stigma in relation to their depression in terms of often feeling discriminated against, misunderstood, and judged by others as a result of public stigma; they discussed internalizing these attitudes. They suggested that a lack of understanding from others, for example from their partners, family, and peers, and unreliable and/or absent support systems resulted in increased feelings of loneliness and social isolation, which reduced the quality and quantity of social relationships. Stigma also reduced their self-esteem and confidence, which in turn fostered secrecy and a reluctance to disclose their depression. The young people, however, also reported having long-term goals and aspirations to reconnect with others. These goals stood in contrast to feeling hopeless and unmotivated during periods of depression. Overall, we reveal how stigma can impact feelings of loneliness, social isolation, and relationships among young people with depression, which could lead to targeted interventions to lessen the impact of stigma in this population.

## Data Availability

Study data are transcripts of interviews containing identifiable information. Data is not publicly available due to concerns that participant privacy may be compromised. Anonymised and de-identified data may be requested from the corresponding author.

## Abbreviations

MDD: Major depressive disorder
MFQ: Mood and Feelings Questionnaire
TA: Thematic analysis
